# Influence of Growth Hormone and Glutamine on Intestinal Stem Cells: A Narrative Review

**DOI:** 10.3390/nu11081941

**Published:** 2019-08-17

**Authors:** Yun Chen, Ya-Hui Tsai, Bor-Jiun Tseng, Sheng-Hong Tseng

**Affiliations:** 1 Department of Surgery, Far Eastern Memorial Hospital, Pan-Chiao, New Taipei 220, Taiwan; 2 Department of Chemical Engineering and Materials Science, Yuan Ze University, Chung-Li, Taoyuan 320, Taiwan; 3 Department of Surgery, National Taiwan University Hospital, Taipei 100, Taiwan

**Keywords:** growth hormone, glutamine, proliferation, differentiation, intestinal stem cells

## Abstract

Growth hormone (GH) and glutamine (Gln) stimulate the growth of the intestinal mucosa. GH activates the proliferation of intestinal stem cells (ISCs), enhances the formation of crypt organoids, increases ISC stemness markers in the intestinal organoids, and drives the differentiation of ISCs into Paneth cells and enterocytes. Gln enhances the proliferation of ISCs and increases crypt organoid formation; however, it mainly acts on the post-proliferation activity of ISCs to maintain the stability of crypt organoids and the intestinal mucosa, as well as to stimulate the differentiation of ISCs into goblet cells and possibly Paneth cells and enteroendocrine cells. Since GH and Gln have differential effects on ISCs. Their use in combination may have synergistic effects on ISCs. In this review, we summarize the evidence of the actions of GH and/or Gln on crypt cells and ISCs in the literature. Overall, most studies demonstrated that GH and Gln in combination exerted synergistic effects to activate the proliferation of crypt cells and ISCs and enhance crypt organoid formation and mucosal growth. This treatment influenced the proliferation of ISCs to a similar degree as GH treatment alone and the differentiation of ISCs to a similar degree as Gln treatment alone.

## 1. Intestinal Stem Cells

**Figure 1:**
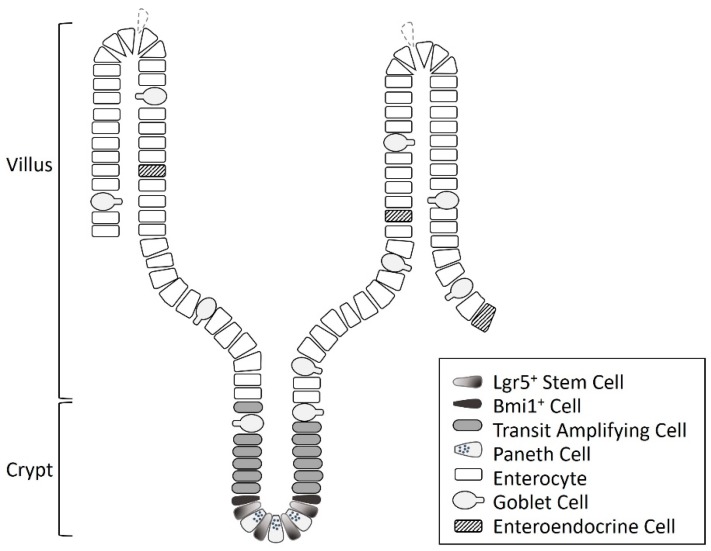
The diagram of the cell types and structures in the intestine.

The intestine comprises mucosa, submucosa, muscle layers, and serosa, as well as intestinal stem cells (ISCs) located at or near the crypt base of the mucosa (Figure 1) [1,2,3]. ISCs play an important role in the growth and the regeneration of the intestinal mucosa and the maintenance of intestinal epithelial homeostasis [4,5,6]. In addition, the recovery of the intestinal mucosa after various insults is highly dependent on the proliferation and the differentiation of ISCs [1,2,7]. The intestinal epithelial cells are renewed every four to five days, and when proliferation is activated, ISCs undergo terminal differentiation as they migrate to the luminal surface [1,2,7,8,9]. They divide to produce transit amplifying cells, which differentiate into absorptive enterocytes and secretory cells, including Paneth cells, goblet cells, and enteroendocrine cells [1,7,8]. Paneth cells move back to the crypt base and intersperse between the stem cells, and the other types of cells migrate into the villi [8,10,11]. In general, absorptive enterocytes constitute about 80% of the small intestinal mucosal epithelial cells, goblet cells 5–10%, enteroendocrine cells 1%, and Paneth cells 5%, respectively [8,12]. ISCs may present various kinds of stemness markers, including leucine-rich repeat-containing G protein-coupled receptor 5 (Lgr5), musashi-1 (Msi1), B cell-specific Moloney murine leukemia virus integration site 1 (Bmi1), and Ephrin receptor-B3 (EphB3) [13,14,15]. These markers can be used to identify the presence or the activation of ISCs [14]. The Lgr5^+^ stem cells, located at the crypt base, are rapidly dividing cells and are important for intestinal renewal [8,13,16,17]. Bmi1^+^ cells, mainly distributed at the +4 position of the crypt, are more quiescent and are activated during stress or injury, producing intestinal progenitor cells to replace the damaged intestinal cells [8,16,17].

In the literature, studies on intestinal stem cells have often investigated the changes in the crypts of the intestines, including crypt cell proliferation, crypt height, crypt volume, crypt depth, and the proliferation index such as Ki67 or proliferating cell nuclear antigen (PCNA) in the crypt region [18,19,20,21,22]. These structural and morphological changes are considered to represent, at least partially, the presence and/or the activity of ISCs [18,19,20,21,22]. In recent years, the isolation of the ISC-containing crypt fraction and observation of crypt organoid (or enteroid) formation in in vitro culture systems has been developed and can be used for the study of intestinal biophysiology, pathology, and regeneration [14,15,23,24,25,26,27,28]. Crypt organoids are a non-transformed tissue culture system containing ISCs and differentiated intestinal epithelial cells within an organotypic three-dimensional structure containing crypt-, villus-, and lumen-like domains [27]. Single Lgr5^+^ stem cell can be cultured into three-dimensional organoids containing all intestinal epithelial cell types of the villi at near-normal ratios [14,15,25,26,29].

The control of intestinal epithelial cell proliferation and differentiation is multifactorial [8]. Since the proliferation and the activity of the ISCs play an important role in growth, regeneration, and integrity of the intestinal mucosa, adequate nutritional supply and energy supplementation are required to support the rapid turnover of ISCs and maintain the integrity of the intestinal epithelium [4,5,6,30]. Furthermore, in clinical settings, patients undergoing extensive resection of the intestine may suffer intestinal failure due to short bowel syndrome (SBS), which results in the significant malabsorption of fluid, electrolytes, and various nutrients [31,32]. After an intestinal resection, structural and functional changes occur in the remaining intestine, including increased wall thickness, mucosal hyperplasia, and mucosal surface area [21,33,34]. Then, the remnant bowel becomes gradually elongated and dilated, which is associated with a slow increase in small bowel absorptive capacity [21,33,34]. The structural and the functional changes in the remaining intestine usually begin on the second post-operative day, after which the intestinal wall width and crypt depth significantly increase as early as the fifth day post operation, which peaks within 1–2 weeks of surgery, the development of which is limited to four weeks after intestinal resection [21,35,36]. However, these changes are generally not enough to support the capacity of the intestine to absorb nutrients in SBS [37]. To meet their nutritional requirements, patients with SBS often require long-term parenteral nutrition (PN) or even total parenteral nutrition (TPN) [22,38]. It is important to determine which constituents specifically affect the activity of ISCs and the intestinal epithelial growth in these patients [30,39]. Various growth factors and nutrients including growth hormone (GH), epidermal growth factor, insulin-like growth factor-1 (IGF-1), caffeic acid, curcumin, bombesin, chitosan, proteins and amino acids such as glutamine (Gln), and long and short chains triglycerides may affect the activity of intestinal stem cells [3,5,8,15,17,26,27,30,33,36,37]. Among them, GH and Gln have been found to activate intestinal epithelial cell proliferation [8,15,27]. However, the effects of GH and Gln on the intestines and the intestinal mucosa are inconsistent across the literature [8,15,27,40], and the influence of GH and/or Gln on the ISCs is unclear. Therefore, in this review, we summarize the evidence for the effect of GH and/or Gln on crypt cells and ISCs in various in vitro and in vivo models and human diseases. We conducted a literature search of PubMed using a combination of the following keywords and their variants: growth hormone, glutamine, intestine, stem cell, and intestinal stem cell (up to 31 May 2019). The search included all articles listed in PubMed. The titles and the abstracts of the identified articles were read, and those concerning growth hormone, glutamine, crypt, intestinal stem cell, and intestinal cell proliferation were included. Only articles written in English were included. Articles involving topics clearly not relevant were excluded. The selected articles were read in full, and further articles identified from their references were also reviewed with a view to include studies that may have been missed in the initial search. A total of 103 references were thus used in the present review. We also discuss the influence of combined GH and Gln on the proliferation and the differentiation of the ISCs and propose possible clinical applications of this combination in intestinal diseases. 

## 2. Growth Hormone and Intestinal Stem Cells

Growth hormone is a pleiotropic hormone that plays an important role in the modulation of various physiological processes [41,42]. It binds to the growth hormone receptor (GHR) to induce intracellular signaling pathways and gene expression [41,42]. GH can promote stem cell activation, proliferation, differentiation, and survival [43]. It exerts anabolic effects largely via the stimulation of IGF-1 production [44]; however, it may also act through IGF-1-independent pathways [42,45,46,47]. GH is also a potent modulator of the hematopoietic system [42,43]. It regulates the proliferation and the differentiation of hematopoietic and immune cells and enhances in vitro erythropoiesis, granulopoiesis, and lymphopoiesis [42,43,48,49,50,51,52]. GH replacement therapy significantly modifies the GHR expression in isolated CD34^+^-enriched hematopoietic progenitor cells, increases clonogenicity of erythroid progenitors, and upregulates cell cycle-propagating proteins, including mitogen-activated protein kinase 1, cyclins D1/E1, PCNA, and IGF-1 in GH deficiency patients [42]. GH also affects the proliferation of neural stem cells and promotes neurogenesis by regulating the proliferation and the differentiation of neural stem cells in the brain [53,54]. 

GH has been found to affect the adaptation of intestinal tissues in animal models and patients with SBS [15,31,33]. Transgenic mice overexpressing GH show an increased body weight and intestinal weight compared to control mice [55]. GH also affects the activity of ISCs or crypt cells (Table 1). GH deficiency following hypophysectomy in rats induces intestinal atrophy, which is attributed largely to a decrease in the mitotic division of epithelial stem cells [18]. Rats genetically deficient in GH show reduced villous volume and surface area, crypt volume, and epithelial cell height in the intestine compared to control rats; intraperitoneal GH administration (1.6 IU/day for 7 d) can increase these values to levels similar to the control [18]. Furthermore, a subcutaneous injection of recombinant bovine GH (1 mg/kg, twice daily for 7 d) in newborn calves was found to reduce villus size, increase crypt depth, and enhance crypt depth/villus height ratio in the small intestine compared to the control [20]. A higher crypt depth and a smaller villus size in GH-treated calves is attributed to an enhanced crypt cell proliferation, the inhibition of crypt cell differentiation, and/or a shortened life span of epithelial cells [20]. In general, the literature shows that GH can increase the crypt depth, indicating increased crypt cell proliferation; however, the effects of GH on villus height are variable.

In the cultured intestinal crypts microdissected from human duodenal biopsied specimens, treatment with GH (0.004 IU/mL) for one day is found to increase crypt epithelial cell proliferation compared to the controls [56]. In addition, the cultured explants of human duodenal mucosa treated with hGH (~1.6 μg/mL; 0.004 IU/mL for 22 h) show an increased number of accumulated metaphase arrests per crypt, indicating increased epithelial crypt cell proliferation [21]. Another study found that treatment with 10 ng/mL GH for 7 days increases the number of cultured mouse intestinal crypt organoids in a Matrigel culture system [15]. In addition, GH increases the expression of stemness markers including Lgr5, Bmi1, Msi1, and EphB3 in cultured mouse crypt organoids compared to the control [15]. GH also affects the expression of the differentiation markers in cultured mouse crypt organoids, decreasing the expression of chromogranin A (a marker for enteroendocrine cell) and increasing the expression of lysozyme (a marker of Paneth cell) and villin (a marker of enterocyte) with no changes in the expression of mucin 2 (a marker for goblet cell) compared to the control [15]. GH increases the Ki67 expression in the crypt organoids, indicating an increased cellular proliferation in the crypt organoids [15]. Further in vivo studies in mice show that the subcutaneous injection of GH (5 mg/kg, once daily for 7 days) increases crypt organoid formation and upregulates the expression of stemness markers including Lgr5, Msi1, and EphB3 but not the expression of Bmi1 [15]. GH injection also affects the expression of differentiation markers. It increases the expression of lysozyme and villin and decreases mucin 2 expression but has no effect on the expression of chromogranin A compared to the control group [15]. These in vitro and in vivo findings indicate that GH treatment activates the proliferation of ISCs, enhances the formation of crypt organoids, increases ISC stemness markers in the intestinal organoids, and drives the differentiation of ISCs into Paneth cells and enterocytes [15]. 

However, several studies report that GH exerts no or mild effects on crypt cells and ISCs [19,37,57]. In newborn piglets with 80% small bowel resection, the subcutaneous injection of GH (0.1 IU/kg/d for 28 days) increases the small bowel lengthening but shows no effects on villus height or diameter, the average number of mitosis per field, the intestinal muscular layer, the wall thickness, or the crypt/villus ratio in the intestine [19]. In 30-day-old suckling rats with 80% midgut bowel resection, the subcutaneous injection of GH (1 mg/kg, every 48 h, eight doses in total) causes intestine lengthening and significantly (but only slightly) increases crypt height and mucosal mass at day 15 post-surgery but not as of day 45 [57]. In aged rats (mean 22 months old) with 80% bowel resection, the subcutaneous injection of GH (1 mg/kg/day for 7 days) enhances the mucosal height and the crypt proliferation in rats fed a high-protein content diet but not in those receiving a normal protein diet [37]. These studies indicate that the age of the animals may affect the response of ISCs or crypt cells to GH (age-related response to GH); that is, age appears to be associated with the effect of GH treatment [57], whereby aged rats require a high protein diet to enhance the effect of GH [37]. 

From these results, it appears that most studies reported that GH can stimulate the proliferation of crypt cells and ISCs with increased crypt depth, enhanced crypt organoid formation, and increased ISC stemness markers in the intestinal organoids. In addition, GH appears to drive the differentiation of ISCs into Paneth cells and enterocytes [8]. GH treatment may need to be supplemented with nutrients, such as a high protein diet, in aged animals [37,57]. This point is particularly important in animals with an intestinal resection of 80% or higher, as the effectiveness of GH treatment may decrease due to a reduced intake or inadequate nutritional absorption in order to promote normal growth [57].

## 3. Glutamine and Intestinal Stem Cells

The rapid turnover of the intestinal mucosa requires a high energy supply. Glutamine, the most abundant amino acid in the serum, is the preferred amino acid for use as a metabolic fuel for enterocytes [8,9,27,58,59,60]. Gln is mainly absorbed by the human jejunum and promotes the growth of the intestinal mucosa, especially in the event of intestinal injury [61]. About 80% of the body’s Gln is contained in skeletal muscle, where the concentration is 15–30 times higher than in the blood plasma [62,63]. Gln is conditionally essential for intestinal homeostasis during catabolic states and plays an important role in various physiological processes, including energy metabolism, peptide and non-peptide synthesis, detoxification of ammonia, systemic acid-base balance, and the immune system [9,27,38,64]. Furthermore, Gln can enhance intestinal mucosal growth, repair, and function, and exerts trophic effects on the intestinal mucosa after small bowel resection or transplant, radiation injury, surgical trauma, ischemic injury, or the administration of cytotoxic drugs [8,9,40,65,66]. Dietary supplementation with Gln has been found to decrease the abundance of Firmicutes, induce a shift of the Firmicutes/Bacteroidetes ratio in favor of Bacteroidetes in the mouse intestines, and activate toll-like receptor 4, pro-inflammatory cytokines, antibacterial substances participating in nuclear factor-κB and c-jun-N terminal kinase signaling pathways, and phosphatidylinositol-3-kinases-Protein kinase B signaling pathways [67]. In addition, Gln can stimulate the secretion of secretory immunoglobulin A in the intestine [68]. These results suggest Gln supplementation alters intestinal bacterial community and activates the innate immunity in the intestine [67,68].

Glutamine has been found to promote the proliferation of intestinal epithelial cell lines, including intestinal porcine epithelial cell line J2 (IPEC-J2) and intestinal porcine epithelial cell line 1 (IPEC-1) [69,70,71]. It stimulates the enterocytes to enter the S-phase of the cell cycle to enhance proliferation [63]. In vitro studies using cell lines have shown that maximal proliferation occurs when the environmental Gln concentration is maintained at 0.5 mM or above, a concentration within the range of normal plasma Gln concentration [72]. The in vivo effects of Gln on intestinal cell proliferation have also been observed in different studies [69,70,71,73,74,75]. Both oral administration and parenteral supplementation of Gln promote the synthesis of DNA and proteins in intestinal epithelial cells, protect the mucosal cells from apoptosis, increase mucosa weight, and prevent mucosal atrophy in animals [38,76]. In rats with orthotopic small bowel isografts, TPN with 2% Gln for 10 days significantly increases mucosal villous height, surface area, and glucose absorption and reduces bacterial translocation compared to the non-Gln TPN group [77]. Similarly, in rats with small bowel transplantation in the form of a Thiry-Vella graft receiving TPN with 2% Gln intravenously or administered directly into the graft for 14 days, Gln supplementation significantly increases mucosal villous height and surface area compared to the controls, with no differences between intravenous injection or direct administration [78]. In weaning piglets, oral Gln (1 g/kg, every 12 h for 14 days) increases the villus height and the villus height/crypt depth ratio from day 3 to day 14 compared to the control piglets [60]. Intermittent supplementation with oral Gln (20% of diet protein) in 27-month-old rats increases the total intestine mass, the villus height, and the difference between villus height and crypt depth but does not change the villous height/crypt depth ratio [61]. Although the results mentioned above show some inconsistences, most studies note that Gln supplementation can increase the villus height in different animal models [60,61,77,78]. The increase in villus height indicates that Gln stimulates villus growth, which may improve nutrient absorption and thus growth performance [60]. 

Glutamine has also been found to exert trophic effects on intestinal stem cells or crypt cells (Table 2) [8,15,27,35,40,62,63,65,66,79,80]. Biopsy samples from normal human ileum, proximal colon, and rectosigmoid incubated with 2 mM Gln for 4 h show increased crypt cell proliferation, with the S-phase labeled with bromodeoxyuridine found mainly in the crypt of the intestine and the colon [63]. Weanling stress may induce villus atrophy, crypt hyperplasia, and increased crypt depth in the intestines of animals [8,65]. In 3-week-old weanling mice, Gln supplementation (10 mg/mL for 2 weeks) in addition to a basal diet and drinking water decreases the crypt depth and increases the villus/crypt ratio but has no effect on villus height in the ileum compared to the control mice [8]. Gln supplementation also significantly increases the number of Ki67-positive cells in each crypt, suggesting that glutamine supplementation promotes crypt cell proliferation in the ileum of weanling mice [8]. In addition, the increased crypt depth in the jejunum resulting from 15 days of malnutrition in rats can be corrected by an oral diet supplemented with 2% Gln for 15 days [65]. In the weaning piglets, a control diet supplemented with 0.5% Gln for 28 days increases the villus height and the crypt depth and decreases the villus/crypt ratio in the ileum [79]. Glutamine also increases the PCNA immunoreactivity in the crypt cells and the number of mitotic mucosal cells [79]. After undergoing extensive small bowel resection, rats with a remnant jejunum of 25 cm administered Gln-enriched diet (12%) for 20 days have a greater villus height in the duodenum mucosa as well as an increased villus height, crypt depth, and thickness of the remnant jejunum mucosa compared to rats administrated a normal control diet [35]. In dogs with 70% small bowel resection, treatment with oral Gln (33 g/5 kg/day) for 15 days increases the villus height and width as well as the crypt depth in the intestinal biopsy samples [40]. Rats fed a 4% or 8% Gln diet for 28 days have a significantly higher mucosal wet weight and a higher protein and DNA content on day 7, as well as a higher average number of mitoses per crypt and a lower villus height and villus/crypt ratio on day 28, than rats fed a glutamine-free isocaloric diet [62]. In rats with resection of 60% of the distal small bowel and an allograft transplantation, continuous infusion of isocaloric polymeric diet with 2% Gln via gastrostomy for 10 days increases the crypt depth compared to the control [66]. However, TPN supplemented with 0.5%, 1.5%, or 2% Gln for six days does not result in any changes in the mitotic activity in the microdissected crypts and has little effect on bromodeoxyuridine labeling in the intestine of rats [80]. As a whole, although the results in the literature are variable, most reports show that Gln supplementation can enhance the proliferation of crypt cells in different animal models [8,40,62,63,65,66,79].

The maximal expansion of the murine crypt enteroid culture derived from the jejunum also requires Gln [27]. Single Lgr5^+^ ISC cannot form crypt buds without Gln, whereas, in the presence of Gln, single ISCs can expand to form organoids [27]. Treatment with 2 mM Gln for 1–4 days promotes epithelial proliferation and crypt expansion in murine enteroids, with the expansion of crypt domains being two- to three-fold greater in the first 48 h in enteroids maintained in Gln than in enteroids maintained in Gln-free medium [27]. Murine enteroids deprived of Gln show a gradual atrophy of crypt-like domains and decreased epithelial proliferation but stable proportions of Paneth and goblet cells at 24 h of Gln deprivation; however, the replenishment of the enteroid medium with Gln restores the epithelial proliferation and promotes crypt regeneration [27]. Furthermore, Gln deprivation beyond 48 h results in the destabilization of the enteroids with progressive crypt atrophy but the persistence of Lgr5^+^ ISCs with the capacity to regenerate enteroids upon Gln rescue [27]. The reintroduction of Gln after 48 h restores the normal levels of proliferation within 3 h and results in the gradual expansion of previously atrophied crypt domains as well as the formation of new crypt domains within 48 h [27]. Treatment with 10 mM Gln for seven days increases the number of cultured crypt organoids and enhances the expression of Msi1 but does not affect the expression of Lgr5, Bmi1, or EphB3 in the crypt organoids [15]. Gln supplementation (10 mg/mL) for two weeks in addition to drinking water and consuming a basal diet in weanling mice promotes the proliferation of intestinal cells and *Lgr5* mRNA expression in the ileum [8]. These findings indicate that Gln can activate intestinal stem cell proliferation, increase crypt organoid formation, and maintain the stability of the crypt organoids [8,15]. However, the effects of Gln on the expression of stem cell markers are not pronounced. Gln deprivation induces a reversible quiescence of ISCs, and Lgr5^+^ ISCs appear resilient to the adverse effects of Gln deprivation, remaining viable but quiescent in culture and reactivating when Gln is reintroduced into the medium [27]. 

Glutamine also influences the differentiation of ISCs. Gln supplementation promotes the expression of α-defensins (a marker for Paneth cells) and C-type lectins (a marker for Paneth cells) in the jejunum and the ileum in mice [67]. Gln also promotes the expression of C-type lectins in the ileum of mice infected with enterotoxigenic *Escherichia coli* [81]. Furthermore, Gln enhances the expression of chromogranin A and mucin 2 in ISCs in vitro, which suggests that Gln drives the differentiation of ISCs into enteroendocrine cells and goblet cells [15]. In in vivo studies, the intraperitoneal injection of Gln (1 g/kg/day) for seven days increases mucin 2 expression and decreases lysozyme expression but has no effect on the expression of chromogranin A or villin in the crypt fractions isolated from the small intestine of mice [15]. These in vitro and in vivo results indicate that Gln stimulates ISCs differentiation into goblet cells and possibly Paneth cells and enteroendocrine cells [15,81]. In contrast, another report found that Gln shows no effect on the number of Paneth cells and goblet cells and does not affect the expression of markers for absorptive enterocytes (Sucrase), Paneth cells (lysozyme and angiogenin 4), goblet cells (mucin 2 and trefoil factor 3), or enteroendocrine cells (chromogranin A and peptide YY) in weanling mice [8]. Gln was also found to have no effects on the expression of Hes1 and mouse atonal homolog 1, which drives intestinal epithelial differentiation into absorptive and secretory lineages [8]. 

As a whole, Gln stimulates villus growth, which may improve nutrient absorption and thus growth performance [60]. Gln influences the proliferation of crypt cells and ISCs, activates epithelial proliferation, and increases crypt organoid formation [8,15,27,35,40,62,63,65,66,79,80]. However, the effects of Gln on the expression of stem cell markers are not prominent, which suggest that the influence of Gln on ISCs is relatively mild [8,15]. In addition, Lgr5^+^ ISCs are resilient to the adverse effects of Gln deprivation, whereby they quiescence in Gln-deprived cultures and reactivate once Gln is resupplied in the medium [27]. As such, Gln mainly functions in the post-proliferation activity of ISCs to maintain the stability of crypt organoids and the intestinal mucosa as well as to stimulate the differentiation of ISCs into goblet cells and possibly also into Paneth cells and enteroendocrine cells [8,15,27,35,40,62,63,65,66,79,80]. However, there is no human study about the effect of glutamine from foods or supplements on the activity of the ISCs. 

## 4. Influence of Combined Growth Hormone and Glutamine on the Intestines

As mentioned above, GH directly stimulates protein synthesis and growth of the intestinal mucosa by increasing cell proliferation and enhancing the absorption of various substances [22,82]. GH also exerts anti-apoptotic and proliferative effects on ISCs [31,38]. Gln is the primary fuel source for enterocytes, which stimulates villus growth, enhances nutrient absorption, regulates stem cell differentiation, and maintains the integrity and the function of the intestinal mucosa [22,31,83,84]. Furthermore, GH increases the luminal uptake of amino acids, including Gln and leucine, from 20% up to 70%, and decreases the intrinsic Gln supply from the degradation of skeletal muscle [36,64,85]. In piglets, the intravenous injection of GH (16 IU) 4 h before the induction of sepsis with *E. coli* increases intestinal Gln uptake compared to the controls and induces the hepatic release of Gln [64]. In an ancillary double-blind, randomized crossover study on the treatment of patients with severe SBS with rhGH (0.05 mg/kg/d) for two 3-week periods, GH was found to enhance the absorption of Gln, the de novo synthesis of Gln, and increased the plasma concentrations of Gln by 17% [86]. Therefore, the combination of GH and Gln (GH+Gln) may induce synergistic effects on the intestine after various insults [22].

GH+Gln has been found to have beneficial effects on muscle protein synthesis, including reducing the negative nitrogen balance, improving immune function, and modulating the inflammatory response of patients with various diseases, including prolonged critical illness after multiple trauma, burns, cystic fibrosis, or following abdominal surgery [87,88,89,90,91]. The effects of GH+Gln in various intestinal disorders in both animals and humans have also been investigated [31,85,92,93]. The combination of a subcutaneous GH injection (1 mg/kg/d) with the oral administration of Gln (1 mg/kg/d) for five days increased the bursting pressure of intestinal anastomosis and improved the safety of anastomosis following intestinal repair in rats with intestinal perforation-induced intra-abdominal sepsis compared to treatment with GH alone, Gln alone, or the control [88]. In addition, after a single intraperitoneal injection of lipopolysaccharide, the rats receiving treatment of GH (intramuscular injection, 2 IU/kg/day) and Gln (10% Gln, 2 mL, every 8 h) showed a lower bacterial colony count and mucosal malondialdehyde levels and higher mucosal glutathione levels in the gut mucosa than control, GH-, or Gln-treated rats [94]. 

In patients with SBS, the combination of GH and Gln, with or without diet modification, has been found to enhance the absorption of nutrients, decrease body fat percentage, improve weight gain and lean body mass (LBM), and reduce the parenteral nutrition requirements, including the PN volume, calories, and infusions [31,93,95]. In rats with 75% intestinal resection and parenteral nutrition, treatment with GH (1 U/kg/day, or 0.33 mg/kg/d) and Gln (2.8%) for six days improved the body weight, the accumulated nitrogen balance, the absolute weight of gastrocnemius muscle, and the weight/length of the remnant small intestine [96]. In addition, GH+Gln increased the PCNA counts by two-fold, decreased the apoptotic index by four-fold, and increased IGF-1 mRNA expression in the jejunal mucosa compared to PN supplementation alone [96]. The subcutaneous injection of GH (0.14 mg/kg/day) combined with a diet rich in Gln for 15 days also improved nitrogen balance and bowel growth but not cell proliferation in rats with 95% small bowel resection [97]. Furthermore, in rats with 85% small bowel resection, a subcutaneous GH injection (0.3 IU, bid) increased body weight, jejuna, and ileal villous height, and mucosal thickness [34]. Compared to the GH group, GH+Gln increased body weight, jejunal and ileal villus height, and mucosal thickness even further [34]. However, Gln supplementation (liquid diet enriched with 20 g Gln) alone did not produce a significant difference in these parameters [34]. In a prospective, double-blind, randomized, placebo-controlled clinical trial in SBS patients, treatment with GH (subcutaneous injection, 0.1 mg/kg per day, four weeks) and Gln (oral, 30 g/day, four months) or GH alone resulted in more weaning from PN than treatment with Gln alone; however, only patients receiving GH+Gln maintained this effect for at least three months [31]. A meta-analysis including 13 trials and involving 258 patients with SBS also demonstrated that GH+Gln with a modified high-carbohydrate-low-fat diet had a positive effect on body weight, stool output, and lean body mass as well as the absorption of carbohydrates, nitrogen, and D-xylose, and weaning off TPN [98].

These results indicate that the effects of combined GH and Gln on various intestinal disorders are inconsistent; however, most of the results demonstrate that GH+Gln exerts a synergistic effect on the intestinal absorption of nutrients, intestinal adaptation, intestinal cell proliferation, general nutritional status, and reduced dependence of parenteral nutrition in SBS patients. In contrast, in rats with 70% jejunoileal resection, the subcutaneous infusion of rat GH (12 mg/kg/day) via an osmotic minipump and Gln (5% weight/weight of the total diet) does not show any enhancement of mucosal mass, mucosal protein, or mucosal DNA levels relative to the control groups 14 days post-enterectomy [99]. 

## 5. Influence of Combined Growth Hormone and Glutamine on Intestinal Stem Cells

The mucosal epithelium of the small bowel undergoes a continuous process of proliferation and differentiation, which are closely related to the activity of the ISCs [31,34,85,92,93,100]. Since both GH and Gln exert a positive effect on the ISCs, and GH+Gln shows a synergistic effect on intestinal cell proliferation in animal studies and in patients with various intestinal disorders [31,38,93,100], it is likely that GH+Gln may also positively affect the intestinal stem cells. However, studies regarding the influence of GH+Gln on ISCs are limited (Table 3). In rats with allogeneic heterotopic small bowel transplantation, villus height, villus width, and crypt depth were found to decrease in the PN control group [82]. Treatment with a subcutaneous injection of GH (1 U/kg/ d) and Gln (2 g/100 mL PN solution) in addition to the nutritional support regimen for 14 days promoted the recovery of graft structure and improved the recipient rats’ protein metabolisms [82]. On the eighth and the fourteenth post-operative day after transplantation, the GH+Gln group showed a greater increase in villus height, villus width, and crypt depth than the GH group, the Gln group, and the control, only with no difference between the villus width of the Gln and the GH+Gln groups [82]. Furthermore, these histologic indices of graft mucosa were nearly restored to preoperative levels in the GH+Gln group [82]. These data indicate GH+Gln exerts synergistic effects on the recovery of the crypt cells of intestinal allografts after small bowel transplantation. 

In rats with 85% mid-small bowel resection, the TPN control group had a lower villus height and mucosal thickness in both the remnant jejunum and the ileum and a shallower jejunal crypt after intestinal resection than the enteral nutrition control group [38]. The subcutaneous injection of GH (0.3 IU, twice daily) combined with an enteral nutrition for eight days improved body mass gain, elevated the plasma IGF-I levels, and increased the villus height and the mucosa thickness in the remnant jejunum and the ileum but did not affect the crypt depth compared to the enteral nutrition control rats [38]. Gln-enriched enteral nutrition (20 g/l) for eight days completely restored the villus height and the mucosal thickness in the ileum and partially restored the villus height, the crypt depth, and the mucosal thickness in the jejunum [38]. However, Gln-enriched enteral nutrition had little effect on body mass and plasma IGF-I levels [38]. Furthermore, Gln-enriched enteral nutrition combined with TPN increased the small intestinal villus height and the mucosa thickness in comparison with the enteral nutrition combined with the TPN control group [38]. Combined treatment with Gln-enriched enteral nutrition (20 g/l), soybean fiber, and GH (0.3 IU, twice daily) for eight days significantly increased the crypt depth in remnant ileum compared to the TPN control group as well as the body mass, the plasma IGF-I levels, the villus height, and the mucosal thickness compared to the enteral nutrition control, the Gln-enriched enteral nutrition, and the enteral nutrition with GH administration [38]. These findings suggest that GH enhances the adaption of the structure of the small bowel after resection but does not affect crypt depth [38]. Although Gln perfusion is beneficial for preserving the structure of the small bowel mucosa and restoring crypt depth during TPN, it has little beneficial effect combined with enteral nutrition (EN) [38]. In addition, GH+Gln combined with soybean fiber can increase the crypt depth of intestinal remnants [38], suggesting GH and Gln may have synergistic effects on crypt cells in SBS. Furthermore, in patients with SBS, treatment with a subcutaneous injection of GH (0.05 mg/kg/day) and orally administered Gln (30 g/day) combined with EN for four weeks increased villus height, Ki67 staining, and crypt depth in the intestinal mucosa compared to pretreatment [22]. In contrast, rats with 80% small bowel resection fed a standard rat chow with a subcutaneous injection of 0.6 IU/day (2 g/day) of GH and 4% Gln for 14 days showed increased intestinal DNA content on the fifth and the fourteenth day post-enterectomy compared to control rats receiving only standard chow, indicating a gain in the cellularity of the gut mucosa [36]. However, the GH+Gln group showed no difference in terms of villus height with the control group, although it did show a reduced wall width and crypt depth [36]. The decreased wall width and crypt depth in the GH+Gln group were not necessarily results from a decreased number of cells and may in fact have been related to the difference of tissue edema [36]. Therefore, GH+Gln is still considered beneficial for the adaptation of the intestine after resection [36]. 

The in vitro effects of GH+Gln on the ISCs have been investigated using cultured intestinal stem cells [15]. Treatment of ISCs with 10 ng/mL GH and 10 mM Gln for seven days resulted in a reduced crypt organoid formation compared to cultured ISCs treated with GH alone; however, no differences were found in terms of organoid formation compared to Gln treatment alone or the control group [15]. In addition, both GH and GH+Gln treatments significantly increased the expression of stemness markers including Lgr5, Bmi1, Msi1, and EphB3; however, GH enhanced the expression of Lgr5 and Msi1 to a higher degree compared to GH+Gln treatment [15]. In contrast, Gln treatment enhanced only the expression of Msi1, but not Lgr5, Bmi1, or EphB3 [15]. These results indicate that the effects of GH+Gln on the proliferation of ISCs are similar to or less than those of GH treatment alone. Furthermore, GH enhanced the expression of the differentiation markers lysozyme and villin, and Gln induced an increased expression of mucin 2 and chromogranin A; on the other hand, GH+Gln increased mucin 2 expression [15]. The expression of the stemness and the differentiation markers in the crypt organoids observed by in situ immunofluorescence staining showed that GH treatment increased the expression of Lgr5, lysozyme, and villin, treatment with Gln strongly enhanced the expression of mucin 2, and GH+Gln resulted in an increased expression of Lgr5 and mucin 2 [15]. In vivo experiments in mice showed that combined GH (subcutaneous injection, 5 mg/kg/day) and Gln (intraperitoneal injection, 1 g/kg/day) treatment for seven days, as well as GH treatment alone, increased crypt organoid formation from cultured ISCs compared to the control or the Gln treatment alone [15]. There was no difference in crypt organoid formation between GH alone and GH+Gln combined treatment [15]. Immunohistochemistry staining also showed a higher Ki67 expression in the crypt region of GH- or GH+Gln-treated mice than that in the control mice [15]. Both GH and GH+Gln treatment significantly upregulated the expression of stemness markers in the crypt fractions of mice. In contrast, Gln treatment only increased Lgr5 expression and had no effect on the expression of Bmi1, Msi1, or EphB3 [15]. In addition, GH significantly increased the expression of lysozyme and vllin in the crypt fractions of mice, whereas Gln increased mucin 2 expression, and GH+GLN had no effect on the expression of the four differentiation markers [15]. These in vivo results indicate that GH+Gln increases the proliferation of ISCs and crypt organoid formation, which is similar to the effects of GH treatment alone [15]. However, GH+Gln did not have any significant effects on the differentiation of the ISCs in vivo. 

Taken together, although the results in the literature are variable, most studies have found that GH+Gln treatment shows synergistic effects on the proliferation of crypt cells and ISCs, an enhancement of the crypt organoid formation, and mucosal growth, and may drive the ISCs to differentiate into goblet cells. Furthermore, GH+Gln is likely to affect the proliferation of ISCs similar to GH treatment alone and influence the differentiation of ISCs in a similar manner as the effects of GLN treatment alone. 

## 6. Factors Affecting the Effects of Growth Hormone and/or Glutamine on the Intestines and Intestinal Stem Cells

The influence of combined GH and/or Gln on crypt cells or intestinal stem cells is related to multiple factors, including dose or concentration, route of administration, treatment duration, the treatment sequence of GH and Gln, in vitro vs. in vivo experiments, culture conditions, animal model, type of animal, age, surgical intervention, additional nutritional supply, timing of the measurement of the experimental parameters, methods used to determine the changes in crypt cells or ISCs, and variable clinical courses of patients in human studies [9,15,22,36,38,82]. Among these factors, dose and duration of treatment are very important and range widely in the literature. The dose may vary up to 5–10-fold, and the duration of treatment varies from 7–28 days. Different routes of administration may also affect the absorption efficiency and the plasma levels of GH and Gln. GH can be administered via intraperitoneal, intramuscular, or subcutaneous injection [15,18,33,93]. Subcutaneous injection is the most commonly used delivery route for GH and often produces a sustained plasma GH concentration in animals and humans [15,93]. Gln can be administered through enteral nutrition, intravenously (such as via total parental nutrition), or through intraperitoneal injection [9,93]. Generally, enteral nutrition is good for prolonged treatment, whereas parental nutrition is superior for achieving target calorie requirements, especially in critically ill patients [9]. However, the absorption of Gln by enterocytes is similar when presented at the basolateral border (i.e., when administered intravenously or intraperitoneally) or at the brush border (i.e., when administered orally) [93]. In the model of intestinal resection, intestinal adaptation occurs early and lasts for an extended period of time [36,57]. The morphological and the proliferative changes that occur after intestinal resection are most prominent 1–2 weeks after bowel resection, after which they return to normal levels [57]. Furthermore, the duration of GH and/or Gln treatments needed in order to induce a positive effect in humans is generally longer than five days [15,31,85,92,93,100]. One study reported that there were no significant changes in the absorption of energy, carbohydrates, electrolytes, or nitrogen after a five day treatment of GH and Gln in humans with SBS [101]. Therefore, a treatment duration of seven days or less is considered a short-term treatment, and the outcome of this treatment may be confounded by the initial potentiation of mucosal adaptation after intestinal resection [36]. Increasing the observation time for the morphological or the structural changes of the intestines to a longer period, such as 1–2 months or more, may more accurately represent the effects of GH and Gln on crypt cells and ISCs. In addition, a small dose over a longer period has been associated with enhanced adaptation [36]. In healthy mice, the absorptive enterocyte, goblet cells, and enteroendocrine cells migrate and differentiate over a period of 2–5 days from the crypt up towards the villous tips; the Paneth cells complete their differentiation and remain within the crypt for around three weeks [102]. This differentiation timeline indicates that ISCs may require the stimulation of GH for their initial proliferation, followed by a relatively long period of Gln exposure for the activation of differentiation [15]. Therefore, the early and the sequential administration of small doses of GH and Gln for a prolonged period of time may be more biologically relevant for the induction of synergistic effects. However, to confirm this hypothesis, future studies will need to carry out well-designed in vivo experiments and well-controlled clinical trials with large population sizes.

## 7. Conclusions

Intestinal stem cells play an important role in growth, recovery, and regeneration of the intestinal mucosa after various insults [1,2,7,30,103]. GH and Gln have been found to exert beneficial effects on the maintenance of the activity and the integrity of ISCs [8,15,27,40,80]. However, the effects of GH and/or Gln on crypt cells and ISCs reported in the literature are inconsistent. GH has been found to increase crypt depth, which suggests it may also enhance crypt cell proliferation; however, reports on the effects of GH on villus height are varied. In addition, GH treatment also requires an adequate supply of nutrients, such as a high protein diet, in order to stimulate the intestinal mucosa in aged animals [37]. This is particularly important in animals with intestinal resection of 80% or higher [37]. Furthermore, GH treatment can activate the proliferation of ISCs, enhance the formation of crypt organoids, increase ISC stemness markers in the intestinal organoids, and drive the differentiation of ISCs into Paneth cells and enterocytes [15]. On the other hand, Gln can stimulate villus growth, which may improve nutrient absorption and thus growth performance [60]. Gln influences the proliferation of crypt cells and ISCs, activates epithelial proliferation, and increases crypt organoid formation; however, the effects of Gln on the expression of stem cell markers in the crypts are mild, which suggests its influence on ISCs is not as strong as GH [8,15]. Gln mainly acts on the post-proliferation activity of ISCs to maintain the stability of crypt organoids and the intestinal mucosa and to stimulate the differentiation of ISCs into goblet cells and possibly Paneth cells and enteroendocrine cells [8,15,27,35,40,62,63,65,66,79,80]. There are few studies reporting the effects of combined GH and Gln on the crypt cells or ISCs, and the results are variable. Most of the studies demonstrate that GH+Gln shows synergistic effects to activate the proliferation of crypt cells and ISCs, to enhance crypt organoid formation, to promote the ISCs to differentiate into goblet cells, and for mucosal growth. This combination treatment appears to affect the proliferation of ISCs in a similar manner as GH treatment alone and influences the differentiation of ISCs in a similar manner as Gln treatment alone.

In summary, both GH and Gln play important roles and exert differential effects in the proliferation and the differentiation of intestinal stem cells [8,27,58,59]. The clinical application of the combination of GH and Gln to stimulate ISCs appears to be reasonable and, based on the data provided by the literature, it could be used to provide effective treatment for various intestinal diseases and to design future studies on ISCs, especially concerning the regulation of their proliferation and differentiation. However, as always, researchers should be cautious when extrapolating findings from in vitro and in vivo studies to clinical settings.

## Figures and Tables

**Table 1:** Effects of growth hormone treatment on intestinal crypt cells and stem cells.

Model	GH Doses	Treatment Duration	Effects	Reference Number
Cultured ISCs from mice	10 ng/ml	7 days	↑Lgr5, Bmi1, Msi1, EphB3, Ki67 ↑lysozyme, villin, ↓chromogranin A 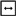 mucin 2 ↑crypt organoids	[15]
Cultured ISCs from GH-treated mice	5 mg/kg/day, sc	7 days	↑Lgr5, Msi1, EphB3 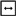 Bmi 1 ↑lysozyme, villin ↓mucin 2 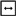 chromogranin A ↑crypt organoids	[15]
Rats genetically deficient in GH	1.6 IU/day, ip	7 days	↑villus volume, surface area, crypt volume, epithelial cell height	[18]
Newborn piglets with 80% intestinal resection	0.1 IU/kg/day, sc	28 days	↑intestinal lengthening 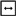 villus height, villus diameter, intestinal muscular layer, wall thickness, mitosis number per filed, crypt/villus ratio	[19]
Newborn calves	1 mg/kg, bid, sc	7 days	↓villus size ↑crypt depth, crypt/villus ratio	[20]
Cultured explants of human duodenal mucosa	0.004 IU/ml	22 h	↑number of metaphase arrests per crypt	[21]
Aged rats with 80% intestinal resection	1 mg/kg/day, sc + high protein diet	7 days	↑mucosal height, crypt proliferation	[37]
Cultured explants of human duodenal mucosa	0.004 IU/ml	1 day	↑crypt cell proliferation	[56]
Suckling rats with 80% intestinal resection	1 mg/kg, sc, qod	16 days (8 doses in total)	↑intestinal lengthening, ↑crypt height, mucosa mass slightly	[57]

ISCs: intestinal stem cells; GH: growth hormone; sc: subcutaneous injection; ip: intraperitoneal injection; im: intramuscular injection; bid: twice a day; qod: every other day; Lgr5: leucine-rich repeat-containing G protein-coupled receptor 5; Msi 1: musashi-1 (Msi1); Bmi 1: B cell-specific Moloney murine leukemia virus integration site 1; EphB3: ephrin receptor-B3 (EphB3); ↑: increased; ↓: decreased; ↔: no change.

**Table 2:** Effects of glutamine treatment on intestinal crypt cells and stem cells.

Model	Gln Doses	Treatment Duration	Effects	Reference Number
3-week-old weaning mice, ileum	10 mg/mL + basal diet	14 days	↓crypt depth ↔villus height ↑villus/crypt ratio ↑Ki67-positive cells in the crypt ↔sucrase, lysozyme, angiogenin 4, mucin 2, trefoil factor 3, peptide YY, chromogranin A,	[8]
Cultured ISCs from mice	10 mM	7 days	↑Msi1 ↔Lgr5, Bmi1, EphB3 ↑mucin 2, chromogranin A ↔lysozyme, villin ↑crypt organoids	[15]
Cultured ISCs from Gln-treated mice	1 g/kg/day, ip	7 days	↑Lgr5 expression ↔Bmi1, Msi 1, EphB3 ↑mucin 2 ↔villin, chromogranin A ↓lysozyme	[15]
Midjejunal crypts of mice	2 mM	1–4 days	↑epithelial cell proliferation, enteroid expansion	[27]
Rats with massive intestinal resection (25 cm jejunum remnant)	12% in diet	20 days	↑villus height, crypt depth, mucosal thickness	[35]
Dogs with 70% intestinal resection	33 g/5 kg/day, oral	15 days	↑villus height and width, crypt depth	[40]
Rats	4% or 8% in diet	28 days	↑mucosal weight, protein and DNA content, mitosis number per crypt ↓villus height, villus/crypt ratio	[62]
Biopsy samples from normal human ileum	Incubation with 2 mM Gln	4 h	↑crypt cell proliferation, BrdU labeling in the crypt	[63]
Rats with 15 days of malnutrition, jejunum	2% in diet	15 days	Correction of malnutrition-induced increased crypt depth	[65]
Rats with 60% intestinal resection + allograft transplantation	2% + isocaloric polymeric diet, infusion via gastrostomy	10 days	↑crypt depth	[66]
Weaning piglets	2% in diet	28 days	↑villus height, crypt depth ↓villus/crypt ratio ↑PCNA staining in crypt cells, number of mitotic mucosal cells, Lgr5 mRNA	[79]
Rats	0.5%, 1%, or 2% in TPN	10 days	↔mitotic activity in the crypts, BrdU labeling	[80]

Gln: glutamine; ip: intraperitoneal injection; bid: twice a day; BrdU: bromodeoxyuridine; PCNA: proliferating cell nuclear antigen; TPN: total parenteral nutrition; Lgr5: leucine-rich repeat-containing G protein-coupled receptor 5; Msi 1: musashi-1 (Msi1); Bmi 1: B cell-specific Moloney murine leukemia virus integration site 1; EphB3: ephrin receptor-B3 (EphB3); ↑: increased; ↓: decreased; ↔: no change.

**Table 3:** Effects of growth hormone and glutamine combined treatment on intestinal crypt cells and stem cells.

Model	GH and Gln Doses	Treatment Duration	Effects	Reference Number
Cultured ISCs from mice	GH: 10 ng/mL Gln: 10 mM	7 days	↔organoid formation ↑Lgr5, Bmi1, Msi1, EphB3 ↑mucin 2 ↔lysozyme, villin, chromogranin A	[15]
Cultured ISCs from GH and Gln-treated mice	GH: 5 mg/kg/day, sc Gln: 1 g/kg/day, ip	7 days	↑organoid formation ↑Ki67 staining ↑Lgr5, Bmi1, Msi1, EphB3 ↔lysozyme, villin, mucin 2, chromogranin A	[15]
Humans with short bowel syndrome	GH: 0.05 mg/kg/day, sc Gln: 30 g/day, enteral	28 days	↑crypt depth, ↑villus height ↑Ki67 staining	[22]
Rats with 80% intestinal resection	GH: 0.6 IU/day (2 g/day), sc Gln: 4%, enteral	14 days	↓crypt depth, ↔villus height ↓intestinal wall width	[36]
Rats with 85% intestinal resection	GH: 0.3 IU, bid, sc Gln: 20 g/l, enteral + soybean fiber	8 days	↑crypt depth, ↑villus height ↑mucosal thickness	[38]
Rats with allogenic heterotopic small bowel transplantation	GH: 1 U/kg/day, sc Gln: 2 g/100 mL parenteral solution	14 days	↑crypt depth, ↑villus height ↑villus width	[82]

GH: growth hormone; Gln: glutamine; sc: subcutaneous injection; ip: intraperitoneal injection; bid: twice a day; Lgr5: leucine-rich repeat-containing G protein-coupled receptor 5; Msi 1: musashi-1 (Msi1); Bmi 1: B cell-specific Moloney murine leukemia virus integration site 1; EphB3: ephrin receptor-B3 (EphB3); ↑: increased; ↓: decreased; ↔: no change.
